# Testing Comparability Between Retrospective Life History Data and Prospective Birth Cohort Study Data

**DOI:** 10.1093/geronb/gbx042

**Published:** 2017-04-21

**Authors:** Stephen Jivraj, Alissa Goodman, George B Ploubidis, Cesar de Oliveira

**Affiliations:** 1 Research Department of Epidemiology and Public Health, University College London; 2 Centre for Longitudinal Studies, UCL Institute of Education, London, UK

**Keywords:** ELSA, Health and economic wellbeing, NCDS, Prospective data, Retrospective data

## Abstract

**Objectives:**

To determine whether comparable prospective and retrospective data present the same association between childhood and life course exposures and mid-life wellbeing.

**Method:**

Prospective data is taken from the 1958 UK National Child Development Study at age 50 in 2008 and earlier sweeps (*n* = 8,033). Retrospective data is taken from the English Longitudinal Study of Ageing at ages 50–55 from a life history interview in 2007 (*n* = 921).

**Results:**

There is a high degree of similarity in the direction of association between childhood exposures that have been prospectively collected in National Child Development Study and retrospectively collected in English Longitudinal Study of Ageing and wellbeing outcomes in mid-life. However, the magnitude of these associations is attenuated substantially by the inclusion of measurements, which are difficult or impossible to capture retrospectively, and are only available in prospective data, such as childhood poverty, cognitive ability, and indices of social and emotional adjustment.

**Discussion:**

The findings on the one hand provide some reassurance to the growing literature using life history data to determine life course associations with later life wellbeing. On the other hand, the findings show an overestimation in the retrospective data, in part, arising from the absence in life history data of childhood measures that are not well suited to retrospective collection.

A life course approach to the study of social epidemiology is becoming a cornerstone of the discipline. There is now a substantial and growing body of literature showing what happens to people in early life matters in terms of their later life health and wellbeing (Kuh, Cooper, Hardy, Richards, & Ben-Shlomo, 2014). This may manifest through critical periods when an exposure will affect an outcome, an accumulation of risk where the longer an exposure is experienced inflates the effect of an outcome, or through a pathway where one outcome affects another. The longitudinal data required to test these theories can take a generation to come to fruition and, because of their cost, are often rationalized or brought to an end before they reach their full potential. A solution to this problem is collecting data about people’s earlier life, retrospectively by asking them to recall their fertility, health, work, partnership, and residential history as well as other information about their circumstances in childhood. Reconstructing life histories has become an important component of the Health and Retirement Study (HRS) family of ageing studies (RAND Corporation, 2011; Smith, 1994) and have expanded the analytical possibilities of later life panel study data (Vanhoutte & Nazroo, 2016). 

There is little evidence demonstrating the expense that the relative ease of retrospective data collection has on the ability to draw empirical support for life course theories. Retrospective data is prone to recall bias because people do not always accurately remember or report what happened to them earlier in life. Studies that have directly tested for recall bias show that it is compounded by the length of time since an event or circumstance and it is also affected by the period during the life course that it occurred (Havari & Mazzonna, 2015; Schroder & Börsch-supan, 2008). Smith (2009) suggests there might also be coloring of responses, where individuals who experience serious adult health problems better remember childhood health problems or remember them worse than they really were. More important is the complexity of a person’s circumstances since the event, the frequency with which an event has occurred and a person’s cognitive ability when asked to recall (Brown, 2013). Asking people to remember what happened to them in childhood when they are in older age is therefore potentially problematic because memory is known to decline in older age (Deary et al., 2009). Moreover, people can only remember what they were aware of in childhood (Hardt & Rutter, 2004). Robins et al. (1985) suggest that adults’ reports of welfare receipt in childhood are often inaccurate because they did not realize their family was claiming benefits.

Discordance between retrospective accounts and prospectively collected data may therefore result from information being sought from different people (e.g., parent in childhood and respondent in later life). Similarly, certain measures of childhood circumstance, such as cognitive ability and behavior that can be collected prospectively, typically cannot be taken retrospectively as individuals are unlikely to know or be able to recall them.

It is rare to be able to directly assess the reliability of retrospective data because comparative prospective data are not usually available on the same sample and confidentiality often restricts linkage to administrative records that might be used to validate such data (Pina-Sánchez, Koskinen, & Plewis, 2014). This makes it difficult to determine to what extent retrospective data can be relied upon to draw accurate conclusions about life course theories. Studies testing retrospectively collected data for internal response consistency when asked at multiple points during older age or external consistency when measured against historic aggregate data are more common. Findings are mixed. Ayalon (2015) finds a high level of inconsistency in reports of negative life events over a 4-year period using HRS. In contrast, Haas (2007) and Haas and Bishop (2010) suggest there is reasonable reliability in reports of childhood health using HRS, the Panel Study of Income Dynamics and the Wisconsin Longitudinal Study. Havari and Mazzonna (2015) compare reports on childhood living conditions in the study of health and retirement in Europe (SHARE) with aggregate data and find the data to be similar.

An alternative approach is to compare like for like samples of prospective and retrospective data by harmonizing applicable measures and testing for comparability in each. This is the main objective of this article and rare example of the approach. We will show, with and without controlling for contemporaneous determinants, to what extent do prospective and retrospective samples show similar associations between life course exposures and mid-life wellbeing using a wide range of outcomes. We use harmonized data from two independent studies, a prospective cohort study, the National Child Development Study (NCDS), and a panel study with a retrospective life history interview, the English Longitudinal Study of Ageing (ELSA). A secondary objective in this article is to determine whether there are characteristics that would be difficult to capture retrospectively that may attenuate the relationship between life course exposures and later life outcomes.

## Methods

### Data

Prospective data comprise information collected before an outcome occurs and respondents are tracked longitudinally. For example, NCDS collected information from the mothers of 17,416 births in the Great Britain in 1958 (Power & Elliott, 2006). The NCDS cohort was retraced at age 7 (1965) and again during childhood at ages 11 (1969) and 16 (1974) when data were collected from parents, schools, and the cohort member. The cohort members were interviewed in adulthood at age 23 (1981), age 33 (1991), age 42 (2000), age 44 (2002 biomedical interview), age 46 (2004), age 50 (2008), and age 55 (2013).

We use data collected at multiple sweeps of NCDS for those living in England. Later life measures are taken at age 50 in NCDS. Adult fertility, work, and partnership histories are taken from all adult sweeps up to and including age 50. Childhood ability and behavioral measures are taken from NCDS at age 11 (Shepherd, 2012; Shepherd, 2013) and other childhood circumstances are measured between birth and age 16.

Retrospective data collection involves looking back in time by asking respondents to recall earlier life events and experiences after an outcome has occurred. ELSA, a panel study of community dwelling adults aged 50 and over, initiated in 2002, asked its respondents aged 50 or over in 2007 to complete a life history interview recording what happened to them before they entered the study (Steptoe, Breeze, Banks, & Nazroo, 2013). It asked about fertility, cohabiting relationships, housing, geographical mobility, employment, health, including childhood health, relationship with parents during childhood, and living situation at age 10 using a life grid (Ward, Medina, Mo, & Cox, 2009).

We match data on later life measures from the third wave of ELSA collected in 2006–2007. We limit our sample to those aged 50–55 to ensure a large enough sample comparable in age and cohort to NCDS. We compared our findings to a sample without the age restriction and the results are similar suggesting sample size does not affect our conclusions. Fertility, work and partnership histories as well as childhood circumstances are taken from the ELSA life history interview. Batty et al (2014) provide a detailed comparison of the life history data available in NCDS and ELSA. A complete list of the questionnaires from each study can be found from: http://www.cls.ioe.ac.uk/ncds; http://www.elsa-project.ac.uk.

### Outcome Measures

We use a range of directly comparable outcomes at age 50–55. These are self-reported general health, quality of life, smoking status, cognitive function, home ownership, pension scheme membership, earnings from employment, and family savings. The justification for these measures is that they are available from both samples. However, they have been shown to be directly or indirectly influenced by earlier life circumstances using both prospective and retrospective data.

General health is measured using a widely validated five-point self-rated scale dichotomized into those that report fair or poor health compared with those that report excellent, very good, or good health (Manor, Matthews, & Power, 2000). To ensure a comparable measure, we use a self-rated health question from 2008 to 2009 wave of ELSA. Schröder (2013) uses SHARELIFE to show how later life poor-rated health is influenced by childhood circumstances, including reporting poor health as a child and having few books in the household. A similar result has been found using prospective data from NCDS, where poor-rated health at age 33 was associated with class at birth (Power, Matthews, & Manor, 1998).

Quality of life is measured using CASP-12, an index specifically developed for older age samples (Wiggins, Netuveli, Hyde, Higgs, & Blane, 2007). It measures quality of life on four domains of control, autonomy, self-realization, and pleasure using a summative index, with values ranging from 0 to 36. The 12-item version has strong psychometric properties in older age samples (Bowling & Stenner, 2010; Kim et al., 2015). The wording of each item is identical in NCDS and ELSA. Blane, Webb, Wahrendorf, & Netuveli (2012) and Wahrendorf and Blane (2015) use prospective and retrospective data, respectively, and find an indirect association between childhood social class and quality of life is explained by labor market disadvantage, which in turn is attenuated by education.

Smoking status is measured by whether a respondent currently smokes cigarettes, asked over two questions in ELSA and one question covering smoking history in NCDS. In a study of six Western countries, Power et al. (2005) find that childhood class measured prospectively is related to current smoking status in midlife. In other European countries where childhood exposures were measured retrospectively, only women from low childhood socioeconomic backgrounds were more likely to smoke.

Cognition is measured using a standardized summary score ranging from 2 to 52, based on an assessment of memory and executive function. The tests were applied in the same way in NCDS and ELSA. High scores indicate better cognitive function. Memory is assessed through a verbal learning and recall test that asks respondents to recall 10 words immediately and after a short delay. Executive function is assessed through verbal fluency task and through a letter cancellation task (Lang et al., 2008). There is a wealth of evidence that supports the notion that childhood social class influences later life cognition when using retrospective recall of early life circumstances, but that it is attenuated by contemporaneous social class and educational attainment (Fors, Lennartsson, & Lundberg, 2009; Luo & Waite, 2005; Turrell et al., 2002).

Home ownership is measured by whether a respondent owned their current occupancy outright or with a mortgage. The question on tenure has the same response options in both studies. Membership of a pension scheme is derived in NCDS by those who report having an employer, private groups personal, stakeholder, retirement annuity or self-invested pension plan and in ELSA by those who report being a member of a pension scheme other than the state pension. Weekly earnings from employment is measured by gross earnings from a current job specified over a period of choice of respondents’ for those working in both NCDS and ELSA. In NCDS, respondents are asked for their gross pay before deductions at the last time they were paid, whereas in ELSA respondents are asked about pay in current job including any overtime or bonuses, but before any deductions for tax, national insurance or pension contributions and union dues. Family savings are derived from the total amount of savings and investments that a respondent and their current partner hold. NCDS respondents are simply asked how much do you and your partner hold in savings and investments. ELSA respondents are asked whether they hold a current account, savings account, tax-free savings and investments, premium bounds, stocks and shares, investment trusts, or bonds and gilts, and then asked the value of each. The measures of weekly earnings and family savings are log transformed in the analysis.

Lersch and Luijkx (2015) use retrospective data from SHARELIFE, showing proxies for family wealth in childhood are independently related to home ownership in later life. Chan and Boliver (2011) use prospective measures of social class to show that the unadjusted probability of home ownership in midlife was more than twice as high in respondents with parents who were in managerial compared with those respondents with parents in manual or unskilled occupations in childhood. Paccagnella and Garrouste (2013) demonstrate how childhood social class is indirectly related to current income through education and income in first job when using data from SHARELIFE. Analysis of SHARELIFE shows that retrospectively reported years spent in employment is strongly predicted of pension income in later life (Möhring, 2015).

### Explanatory Variables

#### Later life control variables

We control for a number of adult circumstances at age 50 in NCDS and age 50–55 in ELSA that are common determinants of the outcomes used in the analysis: sex, adult social class, couple status, and education (Chandola & Jenkinson, 2000; Hall, 2006). The measures are directly comparable in the two datasets. Adult social class is measured using the National Statistics Socioeconomic Classification that is determined by the current occupation of a respondent. The occupational categories are managerial and professional, intermediate and routine, and manual. Couple status is derived by whether someone is cohabiting or not. Education is measured by NVQ level and split into three groups: none, some, and degree.

#### Childhood and life course exposures

Some childhood and life course exposures are more likely to be affected by recall bias than others because of their salience. For example, one would expect an individual to recall how many children they have ever had but find it more difficult to recall all the periods of employment, especially if they have experienced repeated periods of unemployment. We therefore expect the estimates of salient life course exposures to be similar in the prospective and retrospective data. The childhood exposures analyzed are social class, presence of hot water in the house, whether the family experienced financial hardship, chronic health conditions, whether parent’s separated and whether in institutional care. The (non-childhood) life course exposures are number of partners ever lived with, proportion of working life in employment and number of living natural children. The main justification for inclusion of these exposures is their comparability of measurement between NCDS and ELSA. Childhood class has been shown to be predictive of later life health and poverty (Government, 2014; Jenkins & Siedler, 2007; Sweeny, 2014). Poor childhood health tends to increase morbidity in later life while controlling for both adult and childhood socioeconomic status (Blackwell, Hayward, & Crimmins, 2001). The influence of parental separation and childhood mistreatment on later life mental and physical health is also thought to directly impact on later life physical and mental health (Edwards, Holden, Felitti, & Anda, 2003; Maier & Lachman, 2000). Multiple partnership separation during the life course has been shown to negatively affect health and wealth (Willitts, Benzeval, & Stansfeld, 2004; Wilmoth & Koso, 2002), especially in women. However, partnership reformation largely offsets this association (Ploubidis, Silverwood, DeStavola, & Grundy, 2015). Being in employment for a longer proportion of one’s working life has been shown to be related to better life health and economic wellbeing, especially in women (Bardasi & Jenkins, 2002; Denton & Boos, 2007). Childlessness and high parity has been shown to be related to poor health and mortality (Grundy & Kravdal, 2010; Read, Grundy, & Wolf, 2011).

Social class in childhood is measured by the father’s occupation in adolescence and split into three categories using the former General Register Office 1970 socio-economic group: non-manual, manual or unclassified job, and out of work. The father’s (or father figure’s) occupation was asked of NCDS respondents’ parents when the respondent was age 16. They were asked to provide a free text answer on the “actual job” and “trade, industry or profession.” If there was a missing value because the respondent did not respond at age 16 or to the specific question, father’s occupation at age 11 is used. In ELSA, father’s (or main carer’s) occupation at age 14 was asked when they entered the study aged 50 or over using the following categories: armed forces, manager, running own business, professional, administrative, skilled, hospitality, sales, plant operator, other job, something else, casual jobs, retired, unemployment, and sick.

Poor childhood health is measured using the presence of at least one of six conditions at or before the age of 16: asthma, bronchitis, severe headaches, epilepsy, emotional problems, or heart problems. In ELSA, each of these is asked separately in the life history interview. In NCDS, they were asked of the parent. Whether a respondent ever had asthma and wheezy bronchitis was asked at age 7, 11, and 16. Headaches in the past year were reported at age 16. Ever having “epileptic attacks” and having previously seen specialist about emotional problems were asked at 11 and 16. The reporting of congenital heart problems at age 7 or heart complaints in the last year were used to derive the presence of heart problems.

The presence of hot water in the household was measured at age 11 in NCDS by asking their parent. They were asked to report sole, shared, or no use of hot water. ELSA respondents were asked to recall whether or not they had hot water in the household they were living when they were aged 10. Whether a respondent’s parents ever permanently separated or divorced during childhood was asked at age 33 in NCDS and in later life in ELSA. Childhood financial hardship was measured in NCDS by asking parents whether the family experienced serious financial hardship in the past 12 months at ages 11 and 16. In ELSA, respondents were asked to retrospectively recall whether they have ever experienced financial hardship and at what age. We create a binary indicator of those that remember experiencing financial hardship by age 16. Whether a person was in institutional care during childhood was measured in NCDS by asking parents when the respondent was aged 11 or 16 whether they were ever in local authority or voluntary care. ELSA respondents were asked whether they have ever lived in a children’s home or been fostered with another family.

The number of partners a person has ever lived with for a month or more was measured using data collected at each adult sweep of NCDS. Respondents were asked to date the start and, if applicable, end of a live-in partnership. ELSA respondents were asked to recall all their cohabiting partnership histories at the life history interview. We group those with three or more partners into one category. The proportion of working life spent in employment was measured by dividing by the number of years since a respondent left full time education. In NCDS, work history information was collected between surveys on the start and end of a respondents’ main job. In ELSA, these data were collected on all jobs lasting 6 months or more. We group respondents into four categories of working life spent in employment: 0–50%, 50–85%, 85–99%, and 100%. The number of natural living children a respondent has was collected at age 50 in NCDS and at wave three (age 50–55) in ELSA. We group those with four or more children into one category.

There are other life course exposures that more difficult to harmonize between NCDS and ELSA that could plausibly be considered to have an association with later life wellbeing, including relationship with parents, age at first birth, causes of partnership dissolution, residential history, periods living outside the UK, activity between periods of employment, social activity, health behaviors, and adult health conditions.

#### Childhood controls

Receipt of free school meals is taken from NCDS at age 11, providing a measure of family poverty. During the 1960s and 1970s, free school meals were provided to children whose families received family income supplement, supplementary benefit, or whose income was below a minimum value on a national scale of income (Bynner & Joshi, 2002).

Childhood ability is measured in NCDS using reading comprehensive, arithmetic, and perceptuo-motor tests carried out with respondents when they were aged 11 (Shepherd, 2012). The reading comprehensive test required respondents to choose from a selection of five words that appropriately completed sentences. There were 35 questions, giving a total score between 0 and 35. The arithmetic test comprised 40 items involving numerical and geometric calculations. One mark was awarded for each correct answer, giving a total score between 0 and 40. The perceptuo-motor test asked respondents to copy six designs twice: a circle, square, triangle, diamond, cross, and star. One mark was awarded for each correct attempt, giving an overall score between 0 and 12.

Childhood behavior is measured using the Bristol Social Adjustment Guides (BSAG) and Rutter Behaviour Scale both at age 11 (Shepherd, 2013). The BSAG is designed to obtain a picture of a respondent’s behavior, assessed by a schoolteacher. The overall scores range from 0 to 70, with higher scores indicating more problem behavior. The Rutter Behaviour Scales provides a combined index of internalizing and externalizing difficulties in children. We use parental assessments with an overall score ranging from 0 to 25, with higher scores indicating more problem behavior.

Other childhood controls are available in one of the studies, but not both. Those that were in NCDS that we chose not to include are numerous given the prospective nature of the study, including household amenities (e.g., a telephone), household characteristics (e.g., language spoken in the household), maternal smoking, parental characteristics (e.g., father’s education), birth weight, and health behaviors (e.g., sleep). There are measure in ELSA that we do not include because there is no clear prospective equivalent to test the comparability including, childhood general health and presence of severe health conditions (e.g., cancer).

### Statistical Analysis

To determine whether, despite similarities and differences in the univariate estimates of outcomes and exposures, there is bias in the independent association of life course and childhood exposures on later life wellbeing we fit regression models. We use linear and logistic regression depending on the nature of the outcome variable, but use the same list of exposure and control variables in every model. The NCDS-ELSA specific models are fitted in three steps. The first step contains childhood exposures only. The second step adds life course exposures. The third step adds adult control variables. The first step of the NCDS model is fitted with and without childhood family poverty, childhood ability and childhood behavior controls to determine whether these characteristics, which cannot be measured retrospectively, attenuate associations between childhood exposures and later life wellbeing. We use multiple imputation by chained equations to take account of item non-response across all explanatory variables in the statistical analysis using five imputed datasets where missing values for the outcome variable were removed from the analysis. The models are fitted using the Stata mi impute command separately for NCDS and ELSA and each outcome variable. The model coefficients are shown in the online supplement.

## Results

Table 1 provides a descriptive comparison of the NCDS and ELSA samples for outcomes and exposures at age 50–55. There are a similar proportion of respondents in NCDS and ELSA who report fair or poor self-rated health (18.3% vs 18.8%), smoking (21.8% vs 23.6%), being an owner-occupier (83.9% vs 82.8%), and being a member of a pension scheme (78.9% vs 77.3%). The mean quality of life score (26.1 vs 25.7) and log family savings (8.3 vs 8.4) were also very similar in the two samples. There were small differences in the mean adult cognition score (27.1 vs 26.6) and log weekly pay (6.1 vs 5.9). There are a lower proportion of women in NCDS compared with ELSA (50.8% vs 55.6%). There are a slightly higher proportion of people working in routine occupation groups in NCDS (39.8% vs 36.7%). A similar proportion of respondents are in a couple (20.5% vs 23.2%) and a higher proportion of respondents in NCDS have a degree level qualification compared with ELSA (35.2% vs 25.4%). 

**Table 1.** Characteristics of the NCDS and ELSA Samples at Age 50–55

Outcome variables at age 50 (NCDS) or 50–55 (ELSA)	NCDS	ELSA	p*
Health			
Fair or poor general health (%)	18.3	18.8	.702
CASP-12—mean (*SD*)	26.1 (5.8)	25.7 (6.2)	.100
Smokes (%)	21.8	23.6	.216
Cognition—mean (*SD*)	27.1 (4.8)	26.6 (5.2)	.010
Economic			
Owner occupier	83.9	82.8	.375
Log gross weekly pay—mean (*SD*)*	6.1 (0.8)	5.9 (0.9)	.000
Log family savings—mean (*SD*)	8.3 (3.3)	8.4 (3.5)	.570
Member of pension scheme (%)	78.9	77.3	
Adult characteristics at age 50 (NCDS) or 50–55 (ELSA)			
Male (%)	49.2	44.4	.006
NS-SEC			.022
Routine or not working (%)	39.8	36.7	
Intermediate (%)	20.1	23.9	
Managerial occupation (%)	40.1	39.5	
Not living in a couple (%)	20.5	23.2	.055
Highest qualification			.000
No qualifications (%)	10.1	15.1	
Some qualifications (%)	54.6	59.5	
Degree qualification (%)	35.2	25.4	
*N*	8,033^a^	921	

^a^Sample size of variables related to employment are limited to X in NCDS and x in ELSA.

*X^2^ test/*t*-test *p* value for categorical/continuous variables comparing respondents in NCDS with ELSA.

Table 2 shows that among the childhood and life course exposures available in both datasets there is a strong similarity, except for the indicators of childhood economic disadvantage and health. Fewer than 1 in 30 of the NCDS sample compared with one in six in the ELSA sample report no hot water in childhood. If we assume the prospective NCDS data is more accurate, it may be the case that people born during a period when this household facility was not universal might find it hard to accurately remember when they first lived in a house with hot water when asked to recall in later life. It appears that people underreport whether their family experienced financial hardship in childhood if ask to recall it retrospectively in later life. One in six of NCDS respondents’ parents report being in financial hardship at ages 11 or 16 compared with 1 in 20 of ELSA respondents, who were asked to recall experiencing financial hardship in childhood. This suggests it might be difficult to assess one’s social position retrospectively against norms of more than 30 years ago or that who you ask matters (i.e., parents in childhood or child in later life). The proportion with a childhood health condition was considerably higher in NCDS compared with ELSA (47.5% vs 19.3%) suggesting recall error in the retrospective data. The proportion of respondents who had fathers working in non-manual occupations (40% vs 39%), parents separating in childhood (7.9% vs 8.1%) and reported being in care (2.2% vs 2.9%) were very similar in NCDS and ELSA.

**Table 2.** Life Course Characteristics of the NCDS and ELSA Samples

Childhood characteristics in NCDS and ELSA	NCDS	ELSA	p*
Socioeconomic group of father at age 16 (NCDS) or 14 (ELSA)			.674
Non-manual childhood class (%)	40	39	
Manual or unclassified job (%)	56.1	57.4	
Retired, unemployed or sick (%)	4	3.6	
No hot water in house at age 10 (ELSA) 11 (NCDS) (%)	3.2	16	.000
Childhood chronic health condition	47.5	19.3	.000
Parents separated between birth and 16 (%)	7.9	8.1	.768
Financial hardship at or before age 16 (%)	16	5.2	.000
Whether in institutional care at or before age 16 (%)	2.2	2.9	.167
Childhood characteristics in NCDS only			
Maths test score at age 11—mean (*SD*)	17.9 (10.3)	—	—
Reading test score at age 11—mean (*SD*)	16.8 (6.1)	—	—
Design test score at age 11—mean (*SD*)	8.5 (1.4)	—	—
BSAG score at age 11—mean (*SD*)	7.6 (8.5)	—	—
Rutter score at age 11—mean (*SD*)	6.2 (3.4)	—	—
Free school meals at age 11 (%)	8.2	—	—
Life course characteristics in NCDS and ELSA			
No. of lifetime partners			.057
0 (%)	5	5.2	
1 (%)	59.7	62.2	
2 (%)	23.7	24	
3+ (%)	11.6	8.6	
% working life employed			.000
0–50%	13.8	11.5	
50–85%	29.7	28.9	
85–99%	27.6	23.8	
100%	28.9	35.8	
Number of natural children			.009
0 (%)	15.8	16.1	
1 (%)	13.2	12.4	
2 (%)	41.4	37.6	
3 (%)	19.3	20.1	
4 (%)	10.3	13.9	
*N*	8,033	921	

*X^2^ test *p*-value comparing respondents in NCDS with ELSA.

There was a similar estimate of the number of lifetime live-in partners and natural children in the NCDS and ELSA samples. NCDS respondents were marginally more likely to have three or more partners (11.6% vs 8.6%) and marginally less likely to have four or more natural children (10.3% vs 13.9%). NCDS respondents were less likely to have spent their entire working life in employment (i.e., 100%) (28.9% vs 35.8%), perhaps reflecting a more precise account of their employment history given the shorter recall period.

Given the clear limitations of comparing the presence of hot water and financial hardship in childhood in the two samples, we do not include these as exposures in the statistical analysis. When included, financial hardship is only significant in the prospective data and presence of hot water is not a significant predictor of any outcome in either the prospective or retrospective sample.

**Figure 1.**
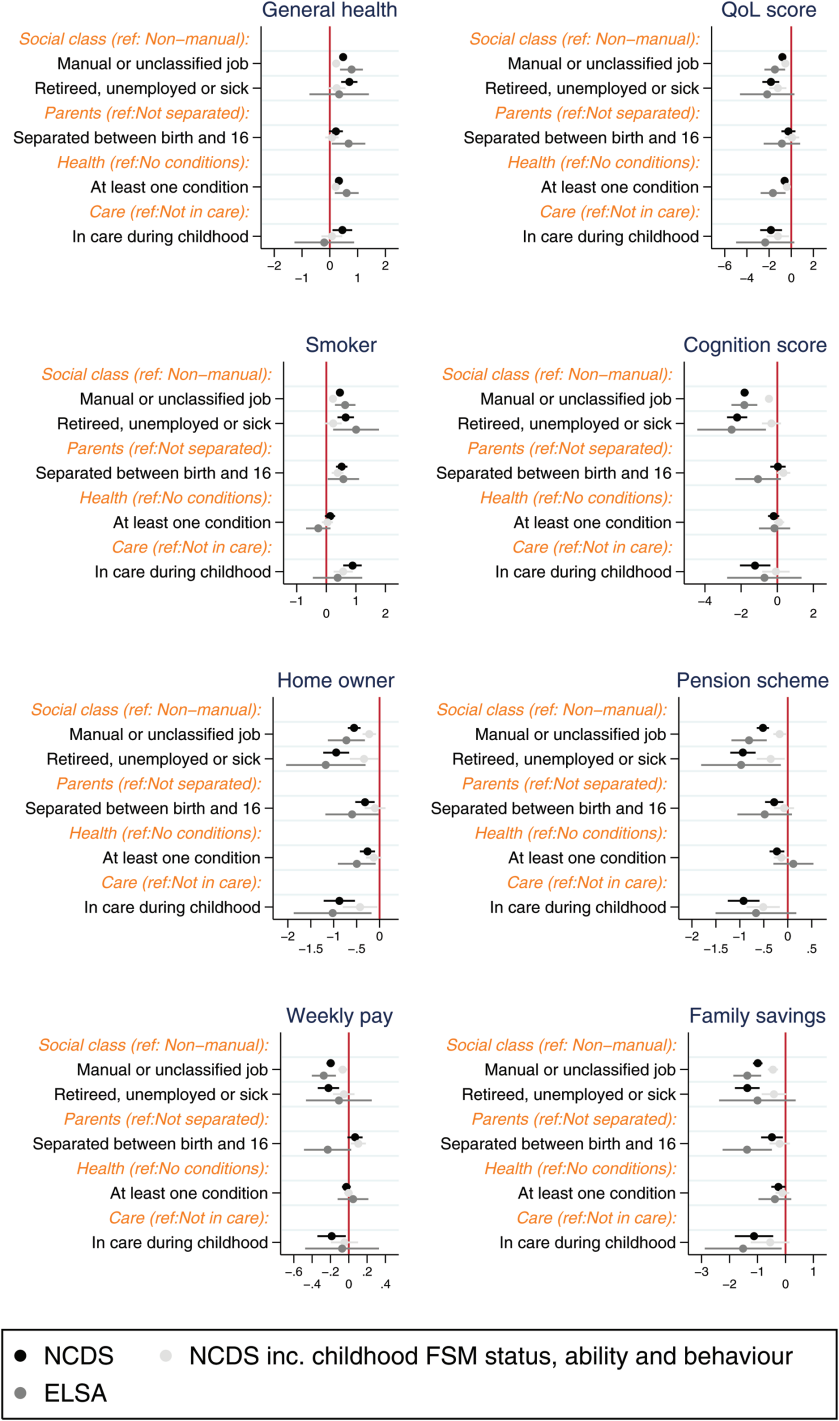
Comparable childhood exposures regressed on mid-life wellbeing outcomes using prospective and retrospective data. Notes: x-axis shows regression coefficients; refers to model 1 in text and appendix.

Figure 1 shows the regression estimates and their 95% confidence intervals for each childhood exposure from model 1. The NCDS estimates are shown, including and excluding, childhood poverty, ability, and behavior controls. Respondents with fathers in a manual social class in childhood were significantly more likely in later life to report poor general health, a lower quality of life score, smoke, a lower cognition score, rent their home, not be a member of a pension scheme, have lower weekly earnings and have lower family savings than those with a father in a non-manual social class. These findings, although in the same direction were of greater magnitude in ELSA compared with NCDS. Moreover, the association was attenuated by more than half for all outcomes, except quality of life, when controlling for childhood poverty, ability and behavior variables in NCDS.

The presence of a child health condition was associated with greater likelihood of poor general health, lower likelihood of home ownership, and lower quality of life scores in both the prospective and retrospective data. However, the magnitude of the estimates is always stronger in ELSA. Poor childhood health is also associated with lower likely of pension membership and family savings in the prospectively data only. This was fully attenuated once taking into account childhood poverty, ability, and behavior. Parental separation was significantly associated with a higher likelihood of smoking, a lower likelihood of being a homeowner and a lower level of family savings in both samples. The strength of association was stronger in ELSA compared with NCDS. The association in NCDS was not significant for home ownership and family savings when taking into account childhood poverty, ability and behavior. Living in institutional care in childhood was significantly associated with all outcomes in NCDS, prior to taking into account childhood controls and often stronger in NCDS than ELSA. Being in care was associated with not being a homeowner and lower family earnings in ELSA. The association was fully attenuated by childhood poverty, ability, and behavior in the self-rated health, cognition, gross weekly pay, and family savings models in NCDS.

**Figure 2.**
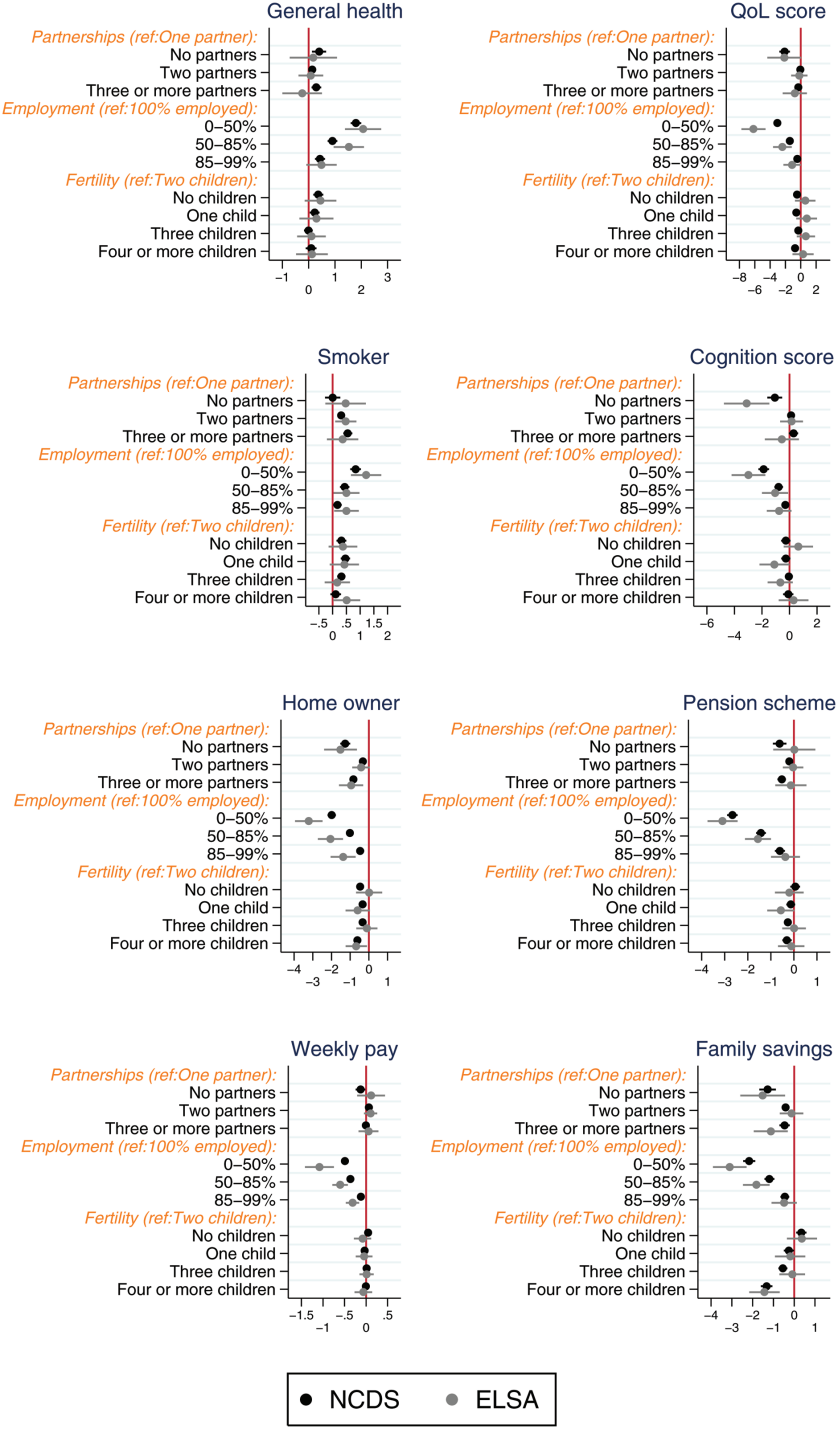
Comparable life course exposures regressed on mid-life wellbeing outcomes using prospective and retrospective data. Notes: includes childhood exposures in Figure 1; x-axis shows regression coefficients; refers to model 2 in text and appendix.

Figure 2 shows the independent estimates of the life course variables when taking into account childhood exposures from the previous step but not including the childhood poverty, ability, or behavior controls. The estimates on each outcome for those who have lived with one partner compared with zero partners or more than one partners is similar in ELSA and NCDS. Cognition is an exception. In ELSA, those who have had one partner reported a cognition value 3.1 lower than others. The estimate, although significant and in the same direction, was −1.1 in the NCDS sample.

Employment history was strongly linearly associated with every outcome in both NCDS and ELSA (i.e., longer period in employment, better wellbeing). The estimates were always stronger in ELSA, especially on quality of life where a respondent working less than 50% of the working life in employment relative to a respondent working all their working life is predicted to have a score 6.2 points lower in ELSA compared to 3.0 points lower in NCDS. Having more than two children relative to two was associated with general health, quality of life, smoking, home ownership, pension membership and family savings in the NCDS sample. In ELSA, having four or more children relative to two is significantly associated with smoking, home ownership and family savings. When significant, the strength of association with fertility history and wellbeing is similar in NCDS and ELSA.

Supplementary Figure 1a and b show the independent estimates for childhood and life course exposures when adding later life controls as well as variables from the previous steps. Their inclusion attenuates the association between many of the childhood and life course exposures and mid-life wellbeing. Childhood social class remains an independent predictor, except on quality of life, owner occupation, weekly earnings and family savings in the ELSA sample, perhaps due to sample size. Child health continues to independently predict poor self-rated health and a lower quality of life, but not home ownership in both NCDS and ELSA. The association with parental separation in childhood was fully attenuated in all models in the ELSA sample, and all but the smoking and weekly earnings models in the NCDS sample. Being in care in childhood remained significantly predictive of quality of life, smoking, and home ownership in NCDS when taking into account adult social class, education level, and couple status. Partnership and fertility history remained stable predictors of mid-life wellbeing where previously significant in the ELSA and, in particular, NCDS samples. The relationship between employment history and each outcome was attenuated but remained predictive of mid-life wellbeing in ELSA and NCDS.

## Discussion

This article has sought to compare retrospectively collected life course exposures that predict mid-life wellbeing with prospectively collected birth cohort data. We have shown similarity in the direction and statistical significance, but not in the magnitude of regression coefficients using retrospective data from the ELSA life history and prospective data from the NCDS cohort. The findings therefore provide some reassurance to the growing literature using life history data to determine life course associations with later life wellbeing, while at the same time highlighting their limitations. These include an overestimation of certain associations and an inability to determine direct association between life course exposures that are difficult or impossible to measure retrospectively. The magnitude of the point estimates tends to differ substantially and from a statistical perspective such that differences could be considered to be indicative of recall bias, assuming that the prospective data used in the study are a “gold standard.”

However, our findings indicate a degree of comparability in salient life events, such as parental separation and being in institutional care in childhood as well as less salient circumstances, such as father’s occupation given estimates of their means and proportions are similar in the comparable prospective and retrospective data. Some measures, however, are not validated and perhaps not appropriate for retrospective collection. These include childhood circumstances that require respondents’ to subjectively measure themselves against historic norms, e.g., the presence of hot water or financial hardship. They have a very different prevalence in the prospective and retrospective data and do not independently predict mid-life wellbeing similarly. This could be in part due to the fact that information was collected from different people (i.e., parents in NCDS and respondents in ELSA), however, this does not appear to affect other measures. There are other measures that do appear to suffer from varying degrees of recall error in the retrospective data, but do not lead to different conclusions with respect to the direction of association in the regression analysis, such as childhood health, employment history, and fertility history.

Measures that aim to determine childhood ability and behavior, or family poverty are not easy to gather retrospectively, at least not directly. These measures predict later life wellbeing over and above comparable childhood exposures and therefore, if they are not taken into account, may lead to an over estimation of the importance of early life characteristics such as childhood social class, for example. They also allow a more direct test of association between later life wellbeing and childhood circumstances that policy makers can act upon. The prospective data also benefit from a wide range of measures collected on the circumstances throughout the life course that not restricted to snapshots at critical periods. Further work using prospective cohort studies should make use of these data as a unique test of life course theories.

There are a number of limitations that the present study should be set against. Not all the data from NCDS is prospective in nature. For example, fertility, partnership, and employment histories are completed by asking respondents to recall what happened between survey interviews. In the case of NCDS, the longest period between interviews for a respondent who completed every sweep is 10 years, between ages 23 and 33. The likelihood of recall error is much smaller than a life history interview at age 50 or older, but probable, especially for people with complicated circumstances. It should also be recognized that prospective data are not necessarily a gold standard. Response inconsistency has been found between sweeps in NCDS even when information derived is from the same source (Brown, 2013). Moreover, we use data on 8,033 respondents to NCDS at 50 when the sample size had reduced in size from 17,416 at birth. Although the sample attrition is known to have been to be systematic to some extent (Hawkes & Plewis, 2006), we accounted for this by employing multiple imputation in both studies assuming a similar bias due to attrition. To relax this assumption we conducted sensitivity analysis in the NCDS where the imputation models were further enriched with auxiliary variables known to be strongly related to missingness since birth (Mostafa & Ploubidis, 2016). This analysis returned similar results with the ones reported in the results section.

The age of respondents in the current study (50–55) is not typically associated with rapid cognitive decline. This often happens at a later age. Asking people aged 70 and over to recall early life events might therefore be more problematic and not lead to the same associations as prospective data, if comparisons were made. Therefore, we cannot generalize our findings to most ageing studies. Further work using earlier born cohorts, such as the 1946 National Survey of Health and Development or the Aberdeen Birth Cohort studies would provide clearer answers on whether these similarities in associations persist in the older old.

We made efforts to ensure comparability between the NCDS and ELSA samples by selecting respondents of similar age at the same point in time. We chose ELSA sample members who responded to an interview between 2006 and 2007 who were born between 1951 and 1956. The NCDS respondents were all born in 1958 and interviewed at age 50 between 2008 and 2009. The sample size from the two studies varied considerably because of these selection criteria. We include 8,033 NCDS respondents and 921 ELSA respondents in our analysis. The comparison of statistical significance between the two studies is dependent on the unbalanced sample size.

In summary, retrospective data do not appear to produce biased estimates with respect to the direction of association between life course exposures and mid-life wellbeing when compared with similar prospective data. There are measures that do not lend themselves to retrospective data collection despite the attempts of survey methodologists, especially measures that require people to compare themselves against historical norms. As we have shown, the inclusion of such measures, if they are available in prospective data, attenuate the relationship between childhood exposures and later life wellbeing, suggesting a potential upward bias in estimates where such measures are not included. We would recommend retrospective life history data limit itself to measures that can be considered objective or salient enough that make it unlikely that survey respondents would misremembered.

## Funding

This work was supported by the Institute of Education/University College London Strategic Partnership Research Development Fund. Goodman and Ploubidis are also supported by the Centre for Longitudinal Studies Resource Centre 2015 - 2020 (ES/M001660/1) and Cross Cohort Research Programme, Employment, Health and Wellbeing (ES/M008584/1).

## Supplementary Material

gbx042_suppl_Supplementary_Appendix
