# Supplementary material for: Testing Comparability Between Retrospective Life History Data and Prospective Birth Cohort Study Data
Source: J Gerontol B Psychol Sci Soc Sci. 2017 Apr 21;75(1):207–17. doi: 10.1093/geronb/gbx042 (PMC6909437; doi:10.1093/geronb/gbx042)
Supplement: gbx042_suppl_Supplementary_Appendix [file gbx042_suppl_supplementary_appendix.docx]

Appendix – Model coefficients

]

| **Self-rated health** |  | NCDS | | | |  | ELSA | | |  |
| --- | --- | --- | --- | --- | --- | --- | --- | --- | --- | --- |
| Variable | Category | Model 1a | Model 1b | Model 2 | Model 3 |  | Model 1 | Model 2 | Model 3 |  |
| Gender (ref: female) | Male | -0.026 | -0.092 | 0.354*** | 0.326*** |  | 0.080 | 0.835*** | 0.932*** |  |
| Childhood social class (ref: non-manual) | Manual or unclassified job | 0.479*** | 0.222** | 0.408*** | 0.247*** |  | 0.778*** | 0.739*** | 0.466* |  |
|  | Retired, unemployed or sick | 0.701*** | 0.249 | 0.526*** | 0.319* |  | 0.334 | 0.292 | 0.036 |  |
| Childhood parental separation (ref: did not separate) | Separated | 0.219 | 0.075 | 0.174 | 0.127 |  | 0.673* | 0.514 | 0.374 |  |
| Childhood health (ref: no chronic condition) | One or more | 0.326*** | 0.209** | 0.219** | 0.225*** |  | 0.608** | 0.606** | 0.699** |  |
| Childhood care (ref: not in institutional care) | In institutional care | 0.450* | 0.073 | 0.205 | 0.066 |  | -0.200 | -0.429 | -0.614 |  |
| Childhood ability and behaviour scores at age 11 | Maths test score |  | -0.0193*** |  |  |  |  |  |  |  |
|  | Reading test score |  | -0.013 |  |  |  |  |  |  |  |
|  | Design test score |  | -0.0610** |  |  |  |  |  |  |  |
|  | BSAG score |  | 0.0210*** |  |  |  |  |  |  |  |
|  | Rutter score |  | 0.0368*** |  |  |  |  |  |  |  |
| Poverty in childhood (ref: no FSM) | Free school meals |  | 0.353** |  |  |  |  |  |  |  |
| No. of lifetime partners (ref: one) | Zero |  |  | 0.395** | 0.075 |  |  | 0.178 | -0.020 |  |
|  | Two |  |  | 0.139 | 0.122 |  |  | 0.084 | 0.052 |  |
|  | Three or more |  |  | 0.293** | 0.287** |  |  | -0.248 | -0.536 |  |
| % Working life employed (ref: 100%) | 0 – 50 % |  |  | 1.797*** | 1.321*** |  |  | 2.069*** | 1.516*** |  |
|  | 50 – 85 % |  |  | 0.905*** | 0.658*** |  |  | 1.527*** | 1.301*** |  |
|  | 85 – 99 % |  |  | 0.432*** | 0.347*** |  |  | 0.489 | 0.425 |  |
| Number of natural children (ref: two) | Zero |  |  | 0.366*** | 0.321** |  |  | 0.451 | 0.419 |  |
|  | One |  |  | 0.228* | 0.159 |  |  | 0.295 | 0.181 |  |
|  | Three |  |  | -0.010 | -0.030 |  |  | 0.107 | 0.172 |  |
|  | Four or more |  |  | 0.093 | 0.026 |  |  | 0.124 | 0.045 |  |
| NSSEC (ref: managerial occupation) | Routine or not working |  |  |  | 0.926*** |  |  |  | 0.707* |  |
|  | Intermediate |  |  |  | 0.207* |  |  |  | -0.021 |  |
|  | Managerial occupation |  |  |  | 0.315*** |  |  |  | 0.521* |  |
| Couple status (ref: no partner) | Living in a couple |  |  |  | 0.477*** |  |  |  | 1.172** |  |
| Qualification (ref: degree) | No qualifications |  |  |  | 0.071 |  |  |  | 0.091 |  |
|  | Some qualifications |  |  |  |  |  | -0.089 | -0.112 | -0.096 |  |
| Age |  | -1.997*** | -1.134*** | -2.992*** | -3.298*** |  | 2.529 | 2.402 | 1.201 |  |
| Constant |  | -0.026 | -0.092 | 0.354*** | 0.326*** |  | 0.080 | 0.835*** | 0.932*** |  |
| Observations |  | 8004 | | | |  | 787 | | |  |

* p<0.05, ** p<0.01, *** p<0.001

| **Quality of life (CASP-12)** |  | NCDS | | | |  | ELSA | | | | |  |
| --- | --- | --- | --- | --- | --- | --- | --- | --- | --- | --- | --- | --- |
| Variable | Category | Model 1a | Model 1b | Model 2 | Model 3 |  | Model 1 | Model 2 | | Model 3 | |  |
| Gender (ref: female) | Male | -0.302* | -0.154 | -0.899*** | -0.839*** |  | 0.323 | -1.028* | | -1.313** | |  |
| Childhood social class (ref: non-manual) | Manual or unclassified job | -0.808*** | -0.527*** | -0.688*** | -0.367* |  | -1.484** | -1.275** | | -0.523 | |  |
|  | Retired, unemployed or sick | -1.846*** | -1.209** | -1.476*** | -1.100** |  | -2.169 | -1.842 | | -1.037 | |  |
| Childhood parental separation (ref: did not separate) | Separated | -0.254 | 0.072 | -0.156 | -0.056 |  | -0.843 | -0.145 | | 0.095 | |  |
| Childhood health (ref: no chronic condition) | One or more | -0.591*** | -0.407* | -0.392** | -0.398** |  | -1.639** | -1.605** | -1.615** | |  |  |
| Childhood care (ref: not in institutional care) | In institutional care | -1.818*** | -1.218* | -1.346** | -1.183* |  | -2.348 | -1.939 | | -1.456 | |  |
| Childhood ability and behaviour scores at age 11 | Maths test score |  | 0.015 |  |  |  |  |  | |  | |  |
|  | Reading test score |  | -0.005 |  |  |  |  |  | |  | |  |
|  | Design test score |  | 0.067 |  |  |  |  |  | |  | |  |
|  | BSAG score |  | -0.0570*** |  |  |  |  |  | |  | |  |
|  | Rutter score |  | -0.0684** |  |  |  |  |  | |  | |  |
| Poverty in childhood (ref: no FSM) | Free school meals |  | -0.967** |  |  |  |  |  | |  | |  |
| No. of lifetime partners (ref: one) | Zero |  |  | -2.104*** | -0.591 |  |  | -2.160 | | -1.005 | |  |
|  | Two |  |  | -0.056 | 0.081 |  |  | -0.191 | | -0.148 | |  |
|  | Three or more |  |  | -0.308 | -0.109 |  |  | -0.782 | | -0.377 | |  |
| % Working life employed (ref: 100%) | 0 – 50 % |  |  | -3.046*** | -1.997*** |  |  | -6.171*** | | -5.041*** | |  |
|  | 50 – 85 % |  |  | -1.405*** | -0.839*** |  |  | -2.389*** | | -1.822** | |  |
|  | 85 – 99 % |  |  | -0.467* | -0.268 |  |  | -1.128 | | -0.837 | |  |
| Number of natural children (ref: two) | Zero |  |  | -0.478* | -0.211 |  |  | 0.585 | | 0.950 | |  |
|  | One |  |  | -0.599** | -0.386 |  |  | 0.769 | | 0.891 | |  |
|  | Three |  |  | -0.322 | -0.313 |  |  | 0.672 | | 0.377 | |  |
|  | Four or more |  |  | -0.741** | -0.688** |  |  | 0.304 | | 0.008 | |  |
| NSSEC (ref: managerial occupation) | Routine or not working |  |  |  | -1.684*** |  |  |  | | -1.190* | |  |
|  | Intermediate |  |  |  | -0.472* |  |  |  | | -1.013 | |  |
|  | Managerial occupation |  |  |  | -1.864*** |  |  |  | | -2.865*** | |  |
| Couple status (ref: no partner) | Living in a couple |  |  |  | -0.528* |  |  |  | | -2.331** | |  |
| Qualification (ref: degree) | No qualifications |  |  |  | -0.392* |  |  |  | | -1.264* | |  |
|  | Some qualifications |  |  |  |  |  | 0.079 | 0.118 | | 0.115 | |  |
| Age |  | 27.09*** | 26.89*** | 28.58*** | 29.16*** |  | 22.81** | 22.60** | | 24.24** | |  |
| Constant |  | -0.302* | -0.154 | -0.899*** | -0.839*** |  | 0.323 | -1.028* | | -1.313** | |  |
| Observations |  | 8004 | | | |  | 787 | | | | |  |

* p<0.05, ** p<0.01, *** p<0.001

| **Current smoker** |  | NCDS | | | |  | ELSA | | | | |  |
| --- | --- | --- | --- | --- | --- | --- | --- | --- | --- | --- | --- | --- |
| Variable | Category | Model 1a | Model 1b | Model 2 | Model 3 |  | Model 1 | Model 2 | | Model 3 | |  |
| Gender (ref: female) | Male | -0.028 | -0.094 | 0.152* | 0.114 |  | -0.265 | 0.006 | | 0.024 | |  |
| Childhood social class (ref: non-manual) | Manual or unclassified job | 0.460*** | 0.226*** | 0.422*** | 0.261*** |  | 0.640*** | 0.627*** | | 0.430* | |  |
|  | Retired, unemployed or sick | 0.655*** | 0.232 | 0.547*** | 0.346* |  | 1.007* | 0.988* | | 0.754 | |  |
| Childhood parental separation (ref: did not separate) | Separated | 0.521*** | 0.380*** | 0.448*** | 0.403*** |  | 0.577* | 0.455 | | 0.367 | |  |
| Childhood health (ref: no chronic condition) | One or more | 0.126 | 0.037 | 0.074 | 0.070 |  | -0.272 | -0.360 | -0.319 | |  |  |
| Childhood care (ref: not in institutional care) | In institutional care | 0.885*** | 0.573*** | 0.767*** | 0.659*** |  | 0.379 | 0.124 | | 0.024 | |  |
| Childhood ability and behaviour scores at age 11 | Maths test score |  | -0.0150*** |  |  |  |  |  | |  | |  |
|  | Reading test score |  | -0.0148* |  |  |  |  |  | |  | |  |
|  | Design test score |  | -0.038 |  |  |  |  |  | |  | |  |
|  | BSAG score |  | 0.0256*** |  |  |  |  |  | |  | |  |
|  | Rutter score |  | 0.005 |  |  |  |  |  | |  | |  |
| Poverty in childhood (ref: no FSM) | Free school meals |  | 0.424*** |  |  |  |  |  | |  | |  |
| No. of lifetime partners (ref: one) | Zero |  |  | 0.000 | -0.622*** |  |  | 0.473 | | -0.021 | |  |
|  | Two |  |  | 0.304*** | 0.285*** |  |  | 0.477* | | 0.440* | |  |
|  | Three or more |  |  | 0.550*** | 0.508*** |  |  | 0.364 | | 0.304 | |  |
| % Working life employed (ref: 100%) | 0 – 50 % |  |  | 0.835*** | 0.382*** |  |  | 1.218*** | | 0.742* | |  |
|  | 50 – 85 % |  |  | 0.439*** | 0.217* |  |  | 0.503* | | 0.293 | |  |
|  | 85 – 99 % |  |  | 0.174* | 0.102 |  |  | 0.503* | | 0.361 | |  |
| Number of natural children (ref: two) | Zero |  |  | 0.320*** | 0.223* |  |  | 0.375 | | 0.130 | |  |
|  | One |  |  | 0.465*** | 0.405*** |  |  | 0.426 | | 0.336 | |  |
|  | Three |  |  | 0.317*** | 0.313*** |  |  | 0.170 | | 0.231 | |  |
|  | Four or more |  |  | 0.111 | 0.094 |  |  | 0.512* | | 0.607* | |  |
| NSSEC (ref: managerial occupation) | Routine or not working |  |  |  | 0.495*** |  |  |  | | 0.304 | |  |
|  | Intermediate |  |  |  | 0.168* |  |  |  | | 0.098 | |  |
|  | Managerial occupation |  |  |  | 0.737*** |  |  |  | | 1.206*** | |  |
| Couple status (ref: no partner) | Living in a couple |  |  |  | 0.828*** |  |  |  | | 1.076*** | |  |
| Qualification (ref: degree) | No qualifications |  |  |  | 0.391*** |  |  |  | | 0.594* | |  |
|  | Some qualifications |  |  |  |  |  | -0.023 | -0.011 | | 0.012 | |  |
| Age |  | -1.697*** | -0.929*** | -2.377*** | -2.773*** |  | -0.313 | -1.894 | | -3.753 | |  |
| Constant |  | -0.028 | -0.094 | 0.152* | 0.114 |  | -0.265 | 0.006 | | 0.024 | |  |
| Observations |  | 8007 | | | |  | 615 | | | | |  |

* p<0.05, ** p<0.01, *** p<0.001

| **Cognition** |  | NCDS | | | |  | ELSA | | | | |  |
| --- | --- | --- | --- | --- | --- | --- | --- | --- | --- | --- | --- | --- |
| Variable | Category | Model 1a | Model 1b | Model 2 | Model 3 |  | Model 1 | Model 2 | | Model 3 | |  |
| Gender (ref: female) | Male | -1.236*** | -1.268*** | -1.616*** | -1.479*** |  | -0.680* | -1.341*** | | -1.504*** | |  |
| Childhood social class (ref: non-manual) | Manual or unclassified job | -1.801*** | -0.461*** | -1.733*** | -1.126*** |  | -1.819*** | -1.731*** | | -0.941** | |  |
|  | Retired, unemployed or sick | -2.215*** | -0.319 | -2.037*** | -1.314*** |  | -2.526** | -2.290* | | -1.446 | |  |
| Childhood parental separation (ref: did not separate) | Separated | 0.041 | 0.347 | 0.014 | 0.134 |  | -1.061 | -0.784 | | -0.454 | |  |
| Childhood health (ref: no chronic condition) | One or more | -0.198 | 0.102 | -0.089 | -0.049 |  | -0.148 | -0.210 | -0.230 | |  |  |
| Childhood care (ref: not in institutional care) | In institutional care | -1.220** | -0.072 | -0.995* | -0.508 |  | -0.713 | -0.138 | | 0.395 | |  |
| Childhood ability and behaviour scores at age 11 | Maths test score |  | 0.137*** |  |  |  |  |  | |  | |  |
|  | Reading test score |  | 0.125*** |  |  |  |  |  | |  | |  |
|  | Design test score |  | 0.047 |  |  |  |  |  | |  | |  |
|  | BSAG score |  | -0.0169* |  |  |  |  |  | |  | |  |
|  | Rutter score |  | -0.012 |  |  |  |  |  | |  | |  |
| Poverty in childhood (ref: no FSM) | Free school meals |  | -0.466* |  |  |  |  |  | |  | |  |
| No. of lifetime partners (ref: one) | Zero |  |  | -1.072*** | -0.609* |  |  | -3.114*** | | -2.684** | |  |
|  | Two |  |  | 0.117 | 0.113 |  |  | 0.151 | | 0.227 | |  |
|  | Three or more |  |  | 0.292 | 0.308 |  |  | -0.552 | | -0.215 | |  |
| % Working life employed (ref: 100%) | 0 – 50 % |  |  | -1.873*** | -0.624** |  |  | -2.981*** | | -1.751** | |  |
|  | 50 – 85 % |  |  | -0.786*** | -0.209 |  |  | -1.052* | | -0.659 | |  |
|  | 85 – 99 % |  |  | -0.295* | -0.129 |  |  | -0.753 | | -0.602 | |  |
| Number of natural children (ref: two) | Zero |  |  | -0.267 | -0.282 |  |  | 0.645 | | 0.413 | |  |
|  | One |  |  | -0.263 | -0.157 |  |  | -1.093* | | -0.945 | |  |
|  | Three |  |  | -0.041 | -0.004 |  |  | -0.675 | | -0.647 | |  |
|  | Four or more |  |  | -0.088 | 0.053 |  |  | 0.299 | | 0.528 | |  |
| NSSEC (ref: managerial occupation) | Routine or not working |  |  |  | -1.430*** |  |  |  | | -1.204** | |  |
|  | Intermediate |  |  |  | -0.621*** |  |  |  | | -0.990* | |  |
|  | Managerial occupation |  |  |  | -0.156 |  |  |  | | -0.124 | |  |
| Couple status (ref: no partner) | Living in a couple |  |  |  | -3.508*** |  |  |  | | -3.592*** | |  |
| Qualification (ref: degree) | No qualifications |  |  |  | -1.735*** |  |  |  | | -2.055*** | |  |
|  | Some qualifications |  |  |  |  |  | 0.177 | 0.154 | | 0.118 | |  |
| Age |  | 28.89*** | 23.19*** | 29.63*** | 30.72*** |  | 18.82** | 21.40** | | 24.97*** | |  |
| Constant |  | -1.236*** | -1.268*** | -1.616*** | -1.479*** |  | -0.680* | -1.341*** | | -1.504*** | |  |
| Observations |  | 7716 | | | |  | 893 | | | | |  |

* p<0.05, ** p<0.01, *** p<0.001

| **Owner occupier** |  | NCDS | | | | | | |  | | ELSA | | | | | |  |
| --- | --- | --- | --- | --- | --- | --- | --- | --- | --- | --- | --- | --- | --- | --- | --- | --- | --- |
| Variable | Category | Model 1a | Model 1b | | Model 2 | | Model 3 | |  | | Model 1 | | Model 2 | | Model 3 | |  |
| Gender (ref: female) | Male | -0.078 | 0.010 | | -0.469*** | | -0.456*** | |  | | 0.246 | | -0.607* | | -0.631* | |  |
| Childhood social class (ref: non-manual) | Manual or unclassified job | -0.553*** | -0.225** | | -0.476*** | | -0.240** | |  | | -0.724*** | | -0.622** | | -0.337 | |  |
|  | Retired, unemployed or sick | -0.946*** | -0.337* | | -0.788*** | | -0.496** | |  | | -1.171** | | -1.185* | | -1.032* | |  |
| Childhood parental separation (ref: did not separate) | Separated | -0.319** | -0.097 | | -0.237* | | -0.116 | |  | | -0.602* | | -0.316 | | -0.085 | |  |
| Childhood health (ref: no chronic condition) | One or more | -0.264*** | -0.128 | -0.110 | | -0.119 | |  | | -0.497* | | -0.442 | | -0.445 | |  |  |
| Childhood care (ref: not in institutional care) | In institutional care | -0.876*** | -0.427* | | -0.546** | | -0.359 | |  | | -1.023* | | -0.695 | | -0.790 | |  |
| Childhood ability and behaviour scores at age 11 | Maths test score |  | 0.0191** | |  | |  | |  | |  | |  | |  | |  |
|  | Reading test score |  | 0.0261** | |  | |  | |  | |  | |  | |  | |  |
|  | Design test score |  | 0.034 | |  | |  | |  | |  | |  | |  | |  |
|  | BSAG score |  | -0.0283*** | |  | |  | |  | |  | |  | |  | |  |
|  | Rutter score |  | -0.0330** | |  | |  | |  | |  | |  | |  | |  |
| Poverty in childhood (ref: no FSM) | Free school meals |  | -0.602*** | |  | |  | |  | |  | |  | |  | |  |
| No. of lifetime partners (ref: one) | Zero |  |  | | -1.263*** | | -0.147 | |  | |  | | -1.520*** | | -0.852 | |  |
|  | Two |  |  | | -0.318*** | | -0.263** | |  | |  | | -0.424 | | -0.390 | |  |
|  | Three or more |  |  | | -0.827*** | | -0.770*** | |  | |  | | -0.952** | | -0.879* | |  |
| % Working life employed (ref: 100%) | 0 – 50 % |  |  | | -1.992*** | | -1.356*** | |  | |  | | -3.214*** | | -2.341*** | |  |
|  | 50 – 85 % |  |  | | -1.019*** | | -0.686*** | |  | |  | | -2.056*** | | -1.648*** | |  |
|  | 85 – 99 % |  |  | | -0.460*** | | -0.339** | |  | |  | | -1.378*** | | -1.161** | |  |
| Number of natural children (ref: two) | Zero |  |  | | -0.466*** | | -0.236* | |  | |  | | 0.010 | | 0.576 | |  |
|  | One |  |  | | -0.331** | | -0.205 | |  | |  | | -0.598 | | -0.424 | |  |
|  | Three |  |  | | -0.334*** | | -0.352*** | |  | |  | | -0.112 | | -0.212 | |  |
|  | Four or more |  |  | | -0.619*** | | -0.682*** | |  | |  | | -0.675* | | -0.861** | |  |
| NSSEC (ref: managerial occupation) | Routine or not working |  |  | |  | | -0.855*** | |  | |  | |  | | -0.961** | |  |
|  | Intermediate |  |  | |  | | -0.353** | |  | |  | |  | | 0.040 | |  |
|  | Managerial occupation |  |  | |  | | -1.540*** | |  | |  | |  | | -1.815*** | |  |
| Couple status (ref: no partner) | Living in a couple |  |  | |  | | -1.139*** | |  | |  | |  | | -1.284** | |  |
| Qualification (ref: degree) | No qualifications |  |  | |  | | -0.350*** | |  | |  | |  | | -0.382 | |  |
|  | Some qualifications |  |  | |  | |  | |  | | -0.012 | | -0.012 | | -0.075 | |  |
| Age |  | 2.255*** | 1.410*** | | 3.689*** | | 4.422*** | |  | | 2.785 | | 5.216 | | 9.436* | |  |
| Constant |  | -0.078 | 0.010 | | -0.469*** | | -0.456*** | |  | | 0.246 | | -0.607* | | -0.631* | |  |
| Observations |  | 8033 | | | | | | |  | | 916 | | | | | |  |

* p<0.05, ** p<0.01, *** p<0.001

| **Pension scheme member** |  | NCDS | | | | | |  | | ELSA | | | | | | | |  | |
| --- | --- | --- | --- | --- | --- | --- | --- | --- | --- | --- | --- | --- | --- | --- | --- | --- | --- | --- | --- |
| Variable | Category | Model 1a | Model 1b | Model 2 | | Model 3 | |  | | Model 1 | | Model 2 | | | Model 3 | | |  |  |
| Gender (ref: female) | Male | 0.931*** | 1.080*** | | 0.439*** | | 0.541*** | |  | | 1.471*** | | 0.740** | | | 0.751** | | |  |
| Childhood social class (ref: non-manual) | Manual or unclassified job | -0.520*** | -0.167* | | -0.417*** | | -0.154* | |  | | -0.807*** | | -0.842*** | | | -0.491* | | |  |
|  | Retired, unemployed or sick | -0.936*** | -0.355* | | -0.714*** | | -0.421** | |  | | -0.976* | | -1.026* | | | -0.601 | | |  |
| Childhood parental separation (ref: did not separate) | Separated | -0.282** | -0.077 | | -0.185 | | -0.134 | |  | | -0.482 | | -0.140 | | | 0.023 | | |  |
| Childhood health (ref: no chronic condition) | One or more | -0.226** | -0.135 | -0.068 | | -0.056 | |  | | 0.120 | | 0.208 | | 0.216 | | |  |  |  |
| Childhood care (ref: not in institutional care) | In institutional care | -0.921*** | -0.511** | | -0.626** | | -0.434* | |  | | -0.664 | | -0.381 | | | -0.394 | | |  |
| Childhood ability and behaviour scores at age 11 | Maths test score |  | 0.0244*** | |  | |  | |  | |  | |  | | |  | | |  |
|  | Reading test score |  | 0.0366*** | |  | |  | |  | |  | |  | | |  | | |  |
|  | Design test score |  | 0.026 | |  | |  | |  | |  | |  | | |  | | |  |
|  | BSAG score |  | -0.0322*** | |  | |  | |  | |  | |  | | |  | | |  |
|  | Rutter score |  | 0.009 | |  | |  | |  | |  | |  | | |  | | |  |
| Poverty in childhood (ref: no FSM) | Free school meals |  | -0.440*** | |  | |  | |  | |  | |  | | |  | | |  |
| No. of lifetime partners (ref: one) | Zero |  |  | | -0.619*** | | -0.231 | |  | |  | | 0.017 | | | 0.485 | | |  |
|  | Two |  |  | | -0.194** | | -0.185* | |  | |  | | -0.038 | | | -0.038 | | |  |
|  | Three or more |  |  | | -0.531*** | | -0.550*** | |  | |  | | -0.133 | | | -0.017 | | |  |
| % Working life employed (ref: 100%) | 0 – 50 % |  |  | | -2.669*** | | -2.146*** | |  | |  | | -3.093*** | | | -2.804*** | | |  |
|  | 50 – 85 % |  |  | | -1.427*** | | -1.136*** | |  | |  | | -1.558*** | | | -1.472*** | | |  |
|  | 85 – 99 % |  |  | | -0.609*** | | -0.515*** | |  | |  | | -0.365 | | | -0.311 | | |  |
| Number of natural children (ref: two) | Zero |  |  | | 0.056 | | 0.098 | |  | |  | | -0.203 | | | -0.236 | | |  |
|  | One |  |  | | -0.139 | | -0.071 | |  | |  | | -0.570 | | | -0.562 | | |  |
|  | Three |  |  | | -0.264** | | -0.248** | |  | |  | | 0.006 | | | -0.091 | | |  |
|  | Four or more |  |  | | -0.303** | | -0.240* | |  | |  | | -0.118 | | | 0.023 | | |  |
| NSSEC (ref: managerial occupation) | Routine or not working |  |  | |  | | -1.103*** | |  | |  | |  | | | -0.349 | | |  |
|  | Intermediate |  |  | |  | | -0.726*** | |  | |  | |  | | | -0.251 | | |  |
|  | Managerial occupation |  |  | |  | | -0.341*** | |  | |  | |  | | | -0.522* | | |  |
| Couple status (ref: no partner) | Living in a couple |  |  | |  | | -1.274*** | |  | |  | |  | | | -2.012*** | | |  |
| Qualification (ref: degree) | No qualifications |  |  | |  | | -0.663*** | |  | |  | |  | | | -0.828** | | |  |
|  | Some qualifications |  |  | |  | |  | |  | | 0.121 | | 0.152* | | | 0.153* | | |  |
| Age |  | 1.434*** | 0.134 | | 2.914*** | | 3.756*** | |  | | -5.067 | | -5.124 | | | -4.288 | | |  |
| Constant |  | 0.931*** | 1.080*** | | 0.439*** | | 0.541*** | |  | | 1.471*** | | 0.740** | | | 0.751** | | |  |
| Observations |  | 8018 | | | | | | |  | | 921 | | | | | | | |  |

* p<0.05, ** p<0.01, *** p<0.001

| **Gross weekly pay (log)** |  | NCDS | | | | | |  | | ELSA | | | | | | | |  | |
| --- | --- | --- | --- | --- | --- | --- | --- | --- | --- | --- | --- | --- | --- | --- | --- | --- | --- | --- | --- |
| Variable | Category | Model 1a | Model 1b | Model 2 | | Model 3 | |  | | Model 1 | | Model 2 | | | Model 3 | | |  |  |
| Gender (ref: female) | Male | 0.691*** | 0.691*** | | 0.557*** | | 0.573*** | |  | | 0.733*** | | 0.443*** | | | 0.395*** | | |  |
| Childhood social class (ref: non-manual) | Manual or unclassified job | -0.198*** | -0.0668** | | -0.181*** | | -0.0583** | |  | | -0.272*** | | -0.256*** | | | -0.049 | | |  |
|  | Retired, unemployed or sick | -0.222*** | -0.054 | | -0.200*** | | -0.092 | |  | | -0.108 | | -0.136 | | | 0.070 | | |  |
| Childhood parental separation (ref: did not separate) | Separated | 0.068 | 0.104* | | 0.072 | | 0.0780* | |  | | -0.231 | | -0.175 | | | -0.199 | | |  |
| Childhood health (ref: no chronic condition) | One or more | -0.026 | -0.003 | -0.010 | | -0.009 | |  | | 0.046 | | -0.025 | | -0.041 | | |  |  |  |
| Childhood care (ref: not in institutional care) | In institutional care | -0.187* | -0.047 | | -0.163* | | -0.036 | |  | | -0.073 | | -0.090 | | | 0.171 | | |  |
| Childhood ability and behaviour scores at age 11 | Maths test score |  | 0.00988*** | |  | |  | |  | |  | |  | | |  | | |  |
|  | Reading test score |  | 0.0193*** | |  | |  | |  | |  | |  | | |  | | |  |
|  | Design test score |  | 0.002 | |  | |  | |  | |  | |  | | |  | | |  |
|  | BSAG score |  | -0.00321* | |  | |  | |  | |  | |  | | |  | | |  |
|  | Rutter score |  | -0.005 | |  | |  | |  | |  | |  | | |  | | |  |
| Poverty in childhood (ref: no FSM) | Free school meals |  | -0.075 | |  | |  | |  | |  | |  | | |  | | |  |
| No. of lifetime partners (ref: one) | Zero |  |  | | -0.130* | | -0.121* | |  | |  | | 0.116 | | | 0.128 | | |  |
|  | Two |  |  | | 0.0666** | | 0.0717** | |  | |  | | 0.105 | | | 0.110 | | |  |
|  | Three or more |  |  | | -0.004 | | -0.002 | |  | |  | | 0.056 | | | 0.131 | | |  |
| % Working life employed (ref: 100%) | 0 – 50 % |  |  | | -0.490*** | | -0.301*** | |  | |  | | -1.083*** | | | -0.757*** | | |  |
|  | 50 – 85 % |  |  | | -0.365*** | | -0.225*** | |  | |  | | -0.604*** | | | -0.490*** | | |  |
|  | 85 – 99 % |  |  | | -0.121*** | | -0.0823*** | |  | |  | | -0.314*** | | | -0.286*** | | |  |
| Number of natural children (ref: two) | Zero |  |  | | 0.048 | | 0.046 | |  | |  | | -0.082 | | | -0.166 | | |  |
|  | One |  |  | | -0.031 | | -0.008 | |  | |  | | -0.045 | | | 0.009 | | |  |
|  | Three |  |  | | 0.010 | | 0.021 | |  | |  | | 0.012 | | | 0.044 | | |  |
|  | Four or more |  |  | | -0.005 | | 0.040 | |  | |  | | -0.063 | | | 0.045 | | |  |
| NSSEC (ref: managerial occupation) | Routine or not working |  |  | |  | | -0.623*** | |  | |  | |  | | | -0.758*** | | |  |
|  | Intermediate |  |  | |  | | -0.465*** | |  | |  | |  | | | -0.468*** | | |  |
|  | Managerial occupation |  |  | |  | | 0.041 | |  | |  | |  | | | 0.002 | | |  |
| Couple status (ref: no partner) | Living in a couple |  |  | |  | | -0.223*** | |  | |  | |  | | | -0.437*** | | |  |
| Qualification (ref: degree) | No qualifications |  |  | |  | | -0.189*** | |  | |  | |  | | | -0.180** | | |  |
|  | Some qualifications |  |  | |  | |  | |  | | -0.036 | | -0.026 | | | -0.033 | | |  |
| Age |  | 5.863*** | 5.294*** | | 6.077*** | | 6.303*** | |  | | 7.615*** | | 7.514*** | | | 8.186*** | | |  |
| Constant |  | 0.691*** | 0.691*** | | 0.557*** | | 0.573*** | |  | | 0.733*** | | 0.443*** | | | 0.395*** | | |  |
| Observations |  | 4982 | | | | | | |  | | 620 | | | | | | | |  |

* p<0.05, ** p<0.01, *** p<0.001

| **Family savings (log)** |  | NCDS | | | | | |  | | ELSA | | | | | | | |  | |
| --- | --- | --- | --- | --- | --- | --- | --- | --- | --- | --- | --- | --- | --- | --- | --- | --- | --- | --- | --- |
| Variable | Category | Model 1a | Model 1b | Model 2 | | Model 3 | |  | | Model 1 | | Model 2 | | | Model 3 | | |  |  |
| Gender (ref: female) | Male | 0.393*** | 0.494*** | | -0.057 | | -0.029 | |  | | 0.461 | | -0.530* | | | -0.577* | | |  |
| Childhood social class (ref: non-manual) | Manual or unclassified job | -0.990*** | -0.448*** | | -0.845*** | | -0.557*** | |  | | -1.365*** | | -1.104*** | | | -0.410 | | |  |
|  | Retired, unemployed or sick | -1.366*** | -0.410 | | -1.009*** | | -0.589** | |  | | -1.004 | | -0.690 | | | -0.170 | | |  |
| Childhood parental separation (ref: did not separate) | Separated | -0.482* | -0.205 | | -0.331 | | -0.260 | |  | | -1.368** | | -0.868* | | | -0.476 | | |  |
| Childhood health (ref: no chronic condition) | One or more | -0.254* | -0.107 | -0.106 | | -0.086 | |  | | -0.378 | | -0.317 | | -0.325 | | |  |  |  |
| Childhood care (ref: not in institutional care) | In institutional care | -1.124*** | -0.547 | | -0.667* | | -0.419 | |  | | -1.511* | | -1.024 | | | -0.792 | | |  |
| Childhood ability and behaviour scores at age 11 | Maths test score |  | 0.0423*** | |  | |  | |  | |  | |  | | |  | | |  |
|  | Reading test score |  | 0.0420*** | |  | |  | |  | |  | |  | | |  | | |  |
|  | Design test score |  | -0.006 | |  | |  | |  | |  | |  | | |  | | |  |
|  | BSAG score |  | -0.0369*** | |  | |  | |  | |  | |  | | |  | | |  |
|  | Rutter score |  | -0.0332* | |  | |  | |  | |  | |  | | |  | | |  |
| Poverty in childhood (ref: no FSM) | Free school meals |  | -0.851*** | |  | |  | |  | |  | |  | | |  | | |  |
| No. of lifetime partners (ref: one) | Zero |  |  | | -1.281*** | | -0.043 | |  | |  | | -1.515** | | | -0.450 | | |  |
|  | Two |  |  | | -0.413*** | | -0.354*** | |  | |  | | -0.130 | | | 0.053 | | |  |
|  | Three or more |  |  | | -0.462*** | | -0.362** | |  | |  | | -1.128** | | | -0.881* | | |  |
| % Working life employed (ref: 100%) | 0 – 50 % |  |  | | -2.166*** | | -1.313*** | |  | |  | | -3.100*** | | | -1.636*** | | |  |
|  | 50 – 85 % |  |  | | -1.193*** | | -0.798*** | |  | |  | | -1.824*** | | | -1.115*** | | |  |
|  | 85 – 99 % |  |  | | -0.449*** | | -0.320** | |  | |  | | -0.484 | | | -0.125 | | |  |
| Number of natural children (ref: two) | Zero |  |  | | 0.341** | | 0.552*** | |  | |  | | 0.374 | | | 0.992** | | |  |
|  | One |  |  | | -0.253* | | -0.100 | |  | |  | | -0.195 | | | 0.037 | | |  |
|  | Three |  |  | | -0.544*** | | -0.546*** | |  | |  | | -0.097 | | | -0.144 | | |  |
|  | Four or more |  |  | | -1.325*** | | -1.309*** | |  | |  | | -1.433*** | | | -1.424*** | | |  |
| NSSEC (ref: managerial occupation) | Routine or not working |  |  | |  | | -0.868*** | |  | |  | |  | | | -0.936*** | | |  |
|  | Intermediate |  |  | |  | | -0.193 | |  | |  | |  | | | -0.125 | | |  |
|  | Managerial occupation |  |  | |  | | -1.454*** | |  | |  | |  | | | -2.331*** | | |  |
| Couple status (ref: no partner) | Living in a couple |  |  | |  | | -1.623*** | |  | |  | |  | | | -2.477*** | | |  |
| Qualification (ref: degree) | No qualifications |  |  | |  | | -0.642*** | |  | |  | |  | | | -0.875** | | |  |
|  | Some qualifications |  |  | |  | |  | |  | | 0.222* | | 0.231** | | | 0.179* | | |  |
| Age |  | 8.913*** | 7.539*** | | 10.14*** | | 10.70*** | |  | | -2.464 | | -1.303 | | | 2.145 | | |  |
| Constant |  | 0.393*** | 0.494*** | | -0.057 | | -0.029 | |  | | 0.461 | | -0.530* | | | -0.577* | | |  |
| Observations |  | 6362 | | | | | | |  | | 785 | | | | | | | |  |

* p<0.05, ** p<0.01, *** p<0.001
